# Whole-Genome Sequence of French Clinical Peptoniphilus catoniae Strain P8546

**DOI:** 10.1128/MRA.01241-19

**Published:** 2019-11-07

**Authors:** Hussein Anani, Issam Hasni, Rita Zgheib, Amael Fadlane, Didier Raoult, Pierre-Edouard Fournier

**Affiliations:** a Institut de Recherche pour le Développement (IRD), Service de Santé des Armées, AP-HM, UMR Vecteurs Infections Tropicales et Méditerranéennes (VITROME), Institut Hospitalo-Universitaire Méditerranée Infection, Aix Marseille Université, Marseille, France; b Institut Méditerranée-Infection, Marseille, France; c Institut de Recherche pour le Développement (IRD), UMR Microbes Evolution Phylogeny and Infections (MEPHI), Institut Hospitalo-Universitaire Méditerranée-Infection, Aix-Marseille Université, Marseille, France; d Special Infectious Agents Unit, King Fahd Medical Research Center, King Abdulaziz University, Jeddah, Saudi Arabia; Broad Institute

## Abstract

In 2016, Peptoniphilus catoniae was reported as a bacterial species isolated from a healthy Peruvian male. In 2018, a clinical strain from the same species was isolated from the stool of a French patient with kidney cancer. The genome of this strain, P8546, was 1,725,465 bp long, with 33.4% G+C content.

## ANNOUNCEMENT

Currently, the genus Peptoniphilus contains 17 species with validly published names that are mainly isolated from human or animal sources (1–7). In 2016, Patel et al. isolated a new bacterial species named Peptoniphilus catoniae from a fecal sample from a healthy Peruvian male (6). In our laboratory, using the culturomics approach (8–10), we isolated another *P. catoniae* strain, P8546, from the feces of a French 70-year-old man suffering from kidney cancer. Bacterial growth was obtained after 24 h of culture in 5% sheep blood-enriched Columbia agar (bioMérieux, Marcy l’Etoile, France) in an anaerobic atmosphere at 37°C. DNA from strain P8546 was extracted using an EZ1 BioRobot with the EZ1 DNA tissue kit (Qiagen). The 16S rRNA gene was amplified using the primer pair fD1 and rP2 (Eurogentec, Angers, France) and sequencing performed using the BigDye Terminator version 1.1 cycle sequencing kit and a 3500xL genetic analyzer capillary sequencer (Thermo Fisher, Saint-Aubin, France), as previously described (11). The 16S rRNA nucleotide sequence was assembled and corrected using the CodonCode Aligner software. This bacterium exhibited 99.40% 16S rRNA sequence similarity with *P. catoniae* strain M6.X2D^T^, its closest phylogenetic neighbor. Then, genomic DNA (gDNA) was sequenced using a MiSeq sequencer (Illumina, Inc., San Diego, CA, USA) with a paired-end strategy. The gDNA was fragmented and amplified by limited-cycle PCR amplification (12 cycles), and we completed the tag adapters and introduced dual-index barcodes following the MiSeq System Denature and Dilute Libraries Guide 15039740-10 (Illumina kit). After purification on AMPure XP beads (Beckman Coulter, Inc., Fullerton, CA, USA), the libraries were then normalized and pooled for sequencing. Automated cluster generation and paired-end sequencing with dual-index reads were performed in a single 39-hour run in 2 × 250-bp format. Total information of 4.76 Gb was obtained from a 242,000/mm^2^ cluster density with a cluster passing quality control filter of 95.83%. Within this run, the index representation of strain P8546 was 12.26%. The 4,572,139 paired-end reads were filtered according to quality using FastQC version 0.11.8 (https://www.bioinformatics.babraham.ac.uk/projects/fastqc/) and trimmed using Trimmomatic version 0.36.6 (12), with default parameters. They were then assembled using the SPAdes version 3.5.0 software (13). The option “careful” was used in order to reduce the number of mismatches and short indels. Default parameters were applied for k values, i.e., k-mer values of 127, 99, 77, 55, 33, and 21. SSPACE (14) and GapFiller (15) were used to combine contigs, using default parameters. The draft genome sequence of *P. catoniae* P8546 is composed of 16 contigs (*N*_50_, 300,781 bp; *L*_50_, 3) and 15 scaffolds (*N*_50_, 428,554 bp; *L*_50_, 2), with 49× coverage, for a total of 1,725,465 bp with 33.4% G+C content. Annotation was performed using Prokka (version 1.12), as previously described (16–18), as follows. Coding sequences were predicted using Prodigal 2.6 (19). ARAGORN 1.2 (20) was used to find tRNA and transfer-messenger RNA (tmRNA) genes, whereas rRNA genes were predicted using Barrnap 0.4. Genome annotation predicted 1,710 genes, including 1,652 protein-coding genes, 1,472 of which (86.1%) were assigned to Clusters of Orthologous Groups. In addition, 58 RNA genes were detected (7 rRNAs, 47 tRNAs, and 4 noncoding RNAs [ncRNAs]). Using the ARG-ANNOT (21) and VFDB (22) databases, no resistance or virulence genes were detected within the genome of *P. catoniae* strain P8546.

### Data availability.

The read sequences and draft genome sequence of *P. catoniae* strain P8546 (BioProject number PRJEB32391 and BioSample number SAMEA5732037) were deposited in GenBank/EBI under accession numbers ERR3393034 and CABDWR000000000, respectively.
